# Supplementary material for: Estimated Costs of the Ipilimumab–Nivolumab Therapy and Related Adverse Events in Metastatic Melanoma
Source: Cancers (Basel). 2022 Dec 21;15(1):31. doi: 10.3390/cancers15010031 (PMC9817856; doi:10.3390/cancers15010031)
Supplement: Supplementary file 1 [file cancers-15-00031-s001.zip › cancers-2063024-supplementary.pdf]

cancers

MDPI

Supplementary File

Estimated costs of the ipilimumab-nivolumab therapy and related adverse events in metastatic melanoma

Table S1. Patient characteristics.

Characteristic	Patients without irAEs due to ipi-nivo	Patients with irAes due to ipi-nivo max grades 1-2	Patients with irAes due to ipi-nivo max grades 3-5
N	8	22	32
Sex-no (%)			
Male	5/8 (62.5%)	15/22 (68%)	17/32 (53%)
Female	3/8 (37.5%)	7/22 (32%)	15/32 (47%)
Age at first diag. (mean, range)	45 (30-60)	53 (19-79)	55 (22-80)
Age at stage IV diag. (mean, range)	48 (33-62)	56 (23-79)	57 (25-80)
Age at start of treatment (mean, range)	48.5 (33-64)	57 (25-79)	58 (27-80)
Melanoma Type (%)			
Cutaneous	5/8 (62.5%)	14/22 (64%)	18/32 (56%)
Unknown primary	1/8 (12.5%)	2/22 (9%)	10/32 (31%)
Mucosal / Conjunctival	0	3/22 (13.5%)	1/32 (3.5%)
Uveal	2/8 (25%)	3/22 (13.5%)	3/32 (9.5%)
Stage at primary diagnosis (AJCC 8th)			
I	2/6 (33%)	4/19 (21%)	1/30 (3.5%)
II	2/6 (33%)	2/19 (10.5%)	9/30 (30%)
III	2/6 (33%)	9/19 (47.5%)	7/30 (23%)
IV	0	4/19 (21%)	13/30 (43.5%)
Unknown	2/8 (25%)	3/22 (13.5%)	2/32 (62.5%)
Breslow (mm)			
< 1	0	3/15 (20%)	2/18 (11%)
< 2	3/4 (75%)	2/15 (13.5%)	6/18 (33.5%)
2 - 4	0	4/15 (26.5%)	6/18 (33.5%)
> 4	1/4 (25%)	6/15 (40%)	4/18 (22%)
Unknown/NA	4/8 (50%)	7/22 (32%)	14/32 (44%)
Ulceration (%)			
Yes	2/3 (66.6%)	6/12 (50%)	8/16 (50%)
No	1/3 (33.3%)	6/12 (50%)	8/16 (50%)
Unknown	5/8 (62.5%)	10/22 (45%)	16/32 (50%)
Previous treatment lines (metastatic setting, %)			
0	6/8 (75%)	16/22 (73%)	28/32 (87.5%)
1	1/8 (12.5%)	4/22 (18%)	3/32 (9%)
2	0	1/22 (4.5%)	0
≥ 3	1/8 (12.5%)	1/22 (4.5%)	1/32 (3.5%)
Previous treatments (metastatic setting, %)			
One prior treatment line			
Immunotherapy	0	2/4 (50%)	1/3 (33.5%)

Targeted Therapy	1/2 (50%)	1/4 (25%)	2/3 (66.5%)
Chemotherapy	0	1/4 (25%)	0
Multiple prior treatment lines			
Immunotherapy	0	1/2 (50%)	1/1 (100%)
Targeted Therapy	0	0	0
Immunotherapy + Chemotherapy	0	0	0
Immunotherapy + Targeted Therapy	1/2 (50%)	0	0
Immunotherapy + Targeted Therapy + Chemotherapy	0	1/2 (50%)	0
Disease Stage (%) at start of ipi-nivo			
unresectable III	1/8 (12.5%)	0	1/31 (32.5%)
IV	7/8 (87.5%)	22/22 100(%)	30/31 (97.5%)
Unknown	0	0	1/32 (3.5%)
N/M Stage (%) at start of ipi-nivo (AJCC 8th)			
III unresectable	1/8 (12.5%)	0	1/31 (3.5%)
M1a	0	1/22 (4.5%)	5/31 (16%)
M1b	1/8 (12.5%)	4/22 (18%)	6/31 (19.5%)
M1c	4/8 (50%)	13/22 (59.5%)	10/31 (32%)
M1d	2/8 (25%)	4/22 (18%)	9/31 (29%)
Number of organs involved at start of ipi-nivo			
< 3 organs	2/8 (25%)	11/22 (50%)	20/32 (62.5%)
≥ 3 organs	6/8 (75%)	11/22 (50%)	12/32 (37.5%)
ECOG at start of ipi-nivo			
0 - 1	1/2 (50%)	11/22 (50%)	15/32 (47%)
> 1	1/2 (50%)	0	0
Unknown	6/8 (75%)	11/22 (50%)	17/32 (53%)
LDH at start of ipi-nivo (%)			
≤ ULN	1/2 (50%)	8/11 (73%)	9/15 (60%)
> ULN	1/2 (50%)	3/11 (27%)	6/15 (40%)
Unknown	6/8 (75%)	11/22 (50%)	17/32 (53%)
Brain metastases (BM) at start of ipi-nivo			
Yes	3/8 (37.5%)	8/22 (36.5%)	11/32 (34%)
No	5/8 (62.5%)	14/22 (63.5%)	21/32 (66%)
Stereotaxic Radiotherapy (SRS) for BM (%)			
Yes	1/3 (33.3%)	6/8 (75%)	9/11 (82%)
No	2/3 (36.6%)	2/8 (25%)	2/11 (18%)
BRAF Mutational status (%)			
BRAF mutant			
V600	6/7 (86%)	11/21 (52.5%)	17/32 (53%)
other BRAF mut	0	1/21 (4%)	3/32 (9%)
No	1/7 (14%)	9/21 (43.5%)	12/32 (38%)
Unknown	1/8 (20%)	1/22 (4.5%)	0
NRAS mutant			
Yes	0	2/20 (10%)	5/30 (17%)

No	6/6 (100%)	18/20 (90%)	25/30 (83%)
Unknown	2/8 (25%)	2/22 (9%)	2/32 (6%)
PDL-1 status			
Positive	1/4 (25%)	6/11 (55%)	11/22 (50%)
Negative	3/4 (75%)	5/11 (45%)	11/22 (50%)
Unknown	4/8 (50%)	11/22 (50%)	10/32 (31%)
Number of cycles ipi-nivo received			
1	6/8 (75%)	0	10/32 (31%)
2	0	3/22 (14%)	7/32 (22%)
3	0	4/22 (18%)	5/32 (16%)
4	2/8 (25%)	15/22 (68%)	10/32 (31%)
Maintenance nivo			
Yes (range of No. of cycles)	2/8 (25%) (10-32)	12/22 (55%) (1-45)	9/32 (28%) (1-32)
No	6/8 (%)	10/22 (45%)	23/32 (72%)

Table S2. RECIST and PERCIST analysis.

Type or response*	RECIST 1.1(%)	PERCIST (%)**
CR	14	19
PR	14	13
SD	14	1
PD	20	17

*Best overall response (BOR)

**12 patients did not have PERCIST analysis (ex: assessment done by CT scan)

% of correlation between PERCIST and RECIST 1.1: 50%

CR: complete response, PR: partial response, SD : stable disease, PD : progressive disease
